# Dataset on significant role of Candesartan on cognitive functions in rats having memory impairment induced by electromagnetic waves

**DOI:** 10.1016/j.dib.2018.11.106

**Published:** 2018-11-26

**Authors:** Mohamad Nasser, Pia Chedid, Ali Salami, Mariam Khalifeh, Said El Shamieh, Wissam H. Joumaa

**Affiliations:** a Rammal Hassan Rammal Research Laboratory, Physio-toxicity (PhyTox) research group, Lebanese University, Faculty of Sciences (V), Nabatieh, Lebanon; b Department of Medical Laboratory Sciences, Faculty of Health sciences, University of Balamand, Beirut, Lebanon; c Department of Medical Laboratory Sciences, Faculty of Health Sciences, Beirut Arab University, Beirut, Lebanon

**Keywords:** Candesartan, Electromagnetic waves, Rats

## Abstract

Rapid growing of mobile phones users has raised about the possible effects of these electromagnetic waves (EMW) on human health. Many studies have examined the role of these EMW on biological systems, but the results are still contradictory and controversial. In addition to EMW, over-activation of angiotensin type 1 receptor (AT1R) has been associated with cognitive decline, incidence and progression of neurodegenerative diseases. Candesartan, an AT1R blocker, is well recognized for treatment of hypertension. However, its role on cognitive functions such as spatial and recognition memory remains elusive. Thus, young rats were divided into 3 groups: control, exposed to radiofrequency electromagnetic waves (EMW), and exposed to EMW during Candesartan treatment (EMW+Cand). Spatial memory performance was assessed using the object recognition test and recognition memory performance using Morris water maze test. Significant differences where found between EMW exposed rats and EMW+Cand exposed rats treated with Candesartan compared to control, EMW group impaired learning, spatial and short term memory along with unaffected sensorimotor function whereas EMW+Cand group improved learning, spatial memory and short term memory deficit induced by EMW in addition to absence of its role on sensorimotor function. Although our data provides evidences of the protective role of Candesartan against EMW-induced cognitive decline, more future studies are still needed to confirm these findings which can provide new fields in treatment of EMW-induced damage by Candesartan.

**Specifications table**

**Table:** 

Subject area	*Biology*
More specific subject area	*Electromagnetic biology and pharmacology*
Type of data	*Figure*
How data was acquired	*Beam balance, Morris maze and Object recognition test*
Data format	*Analyzed*
Experimental factors	*Brief description of any pretreatment of samples*
Experimental features	*Rat animal model along with behavioral tests*
Data source location	*Nabatieh, Lebanon*
Data accessibility	http://dx.doi.org/10.17632/45v3f48cnm.1
Related research article	*Tota S. et al. Candesartan improves memory decline in mice: involvement of AT1 receptors in memory deficit induced by intracerebral streptozotocin. Behav. Brain Res. 2009; 199(2):235–40.*

**Value of the data**

• Our data can be used to indicate the threshold of EMV needed to impair short-term recognition memory in rats.
• Our data can be used to report the time of exposure to EMW needed to disturb the cognitive performance in Sprague Dawley rats.
• Candesartan improves cognitive functions after memory impairment induced by electromagnetic waves in Sprague Dawley rats.

## 1 Data

**Fig. 1:**
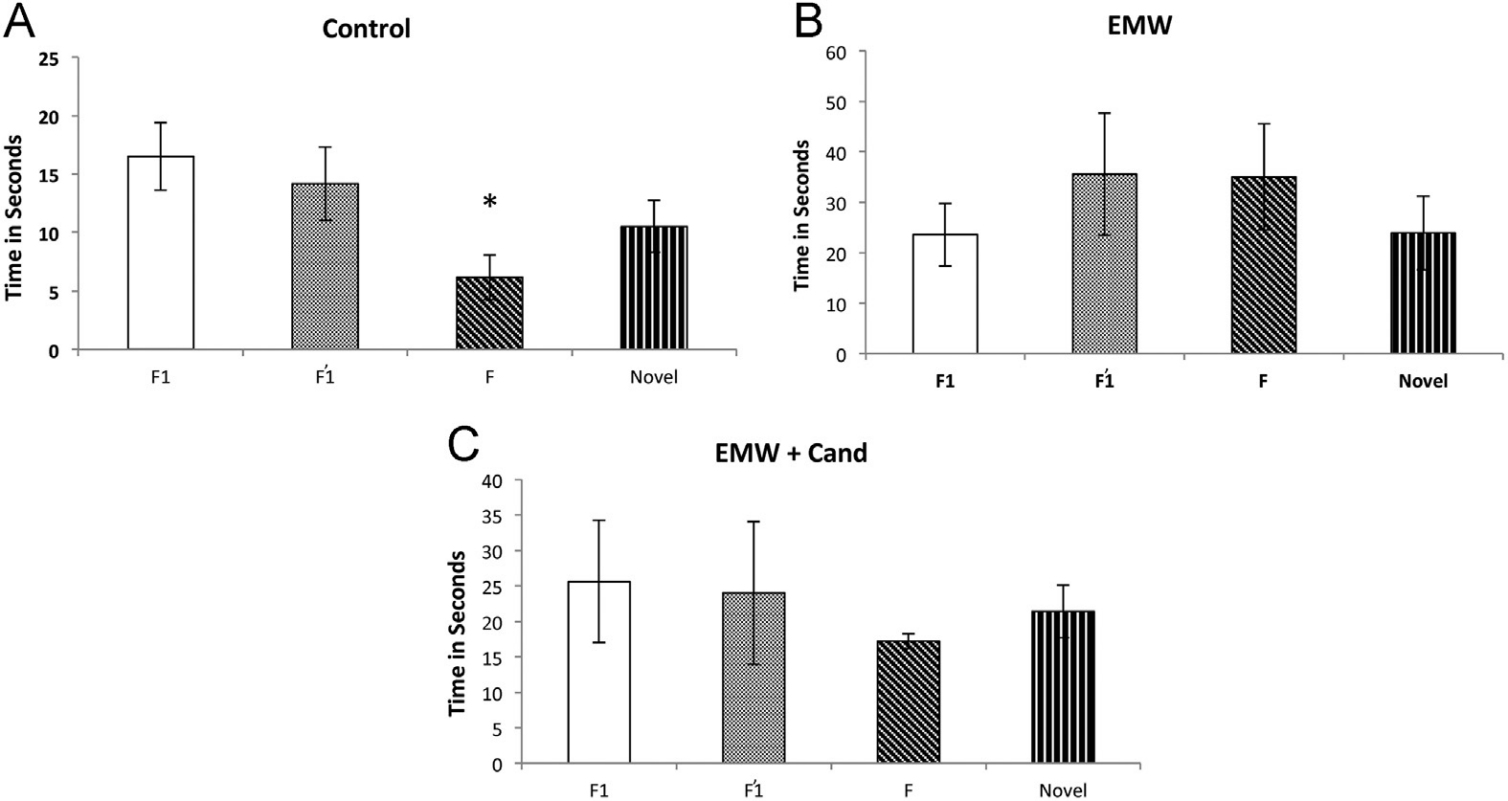
Object recognition test.

Fig. 1-A show no deficit in short term memory in the control non-exposed group of rats as they succeed in spending significantly greater (*p*<0.05) time in exploring the novel object during the test phase. In contrast, the EMW group in Fig. 1-B, show young rats spending less time in exploring the novel object compared to the familiar one (F, F1, and F’1). However, rats treated with Candesartan along with their exposure to EMW Fig. 1-C show increased time in exploring novel object compared to familiar.

**Fig. 2:**
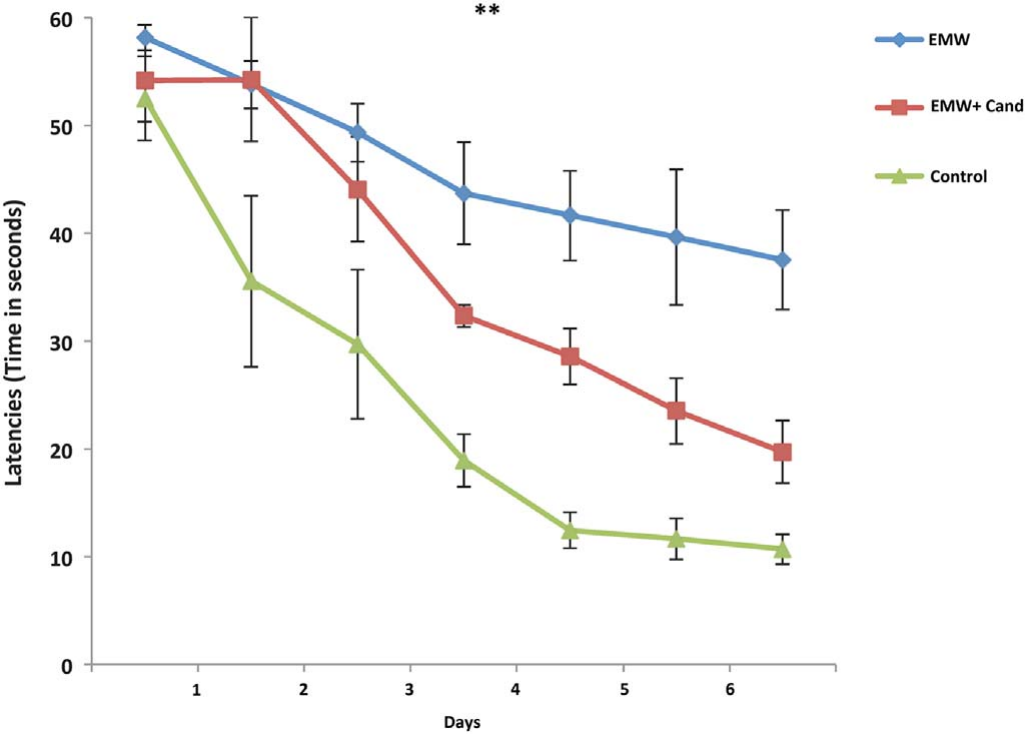
Average escape latencies during the training session (consisted of 7 trials) of control group (n=6), EMW group (n=11) and EMW+Cand group (n=5).

The change in performance was obvious between the groups (Fig. 2). All the three groups start their performance approximately in the same range (52–60 s); there was no difference in the latencies to find the hidden platform. The performance of the control rats was improved greatly and progressively compared to the other groups for which the latency to reach the platform decreased rapidly through the seven days of the experiment, in day 7 the rats reached the platform in ~11 s. However, the slower rate of performance was observed in EMW group and the treated group shows a moderate decrease in latencies stands approximately in the middle between EMW and control group. The data revealed a significant difference (***P<0.001) between EMW and control groups, significant difference (††*P*<0.01) between EMW and EMW+Cand groups in addition to significant difference (##*P*<0.01) in latencies between treated and control groups Exposure to the EMW impaired overall performance (learning and spatial memory); the exposed groups did not finally reach the same level of accuracy as the control group.

The data for recognition of novel objects were presented as the mean ± standard error 1-A: Control group (*n*=6); 1-B: EMW group (*n*=11); 1-C: EMW+Cand group (*n*=5).

All rats were trained for three trials per day for 7 days. ***P<0.001 significant difference between EMW and control. ##*P*<0.01 significant difference between EMW+Cand and control. ††*P*<0.01 significant difference between EMW and EMW+Cand.

## 2 Experimental design, materials, and methods

All procedures in this article were performed in accordance with the stipulation of the Helsinki declarations, and comply with the ARRIVE guidelines and carried out in accordance with the U.K. Animals.

### 2.1 Rat model

Twenty-two young male Spargue-Dawley rats (3 weeks age) were housed in plexi cages inside a well-ventilated room maintained at 23 ± 1 °C with alternating one cycle of 12-h light: 12-h darkness. The animals had access to a standard diet and water and were divided into 3 groups: control group (*n*=6; not exposed to RF-EMW), EMW (exposed group, *n*=11) and EMW+Cand (exposed treated with Candesartan, *n*=5). Candesartan cilexetil was purchased from Takeda pharmaceutical company limited. 0.5 mg /kg per day of Candesartan tablet was administered through drinking water and supplied to rats instead of pure water for 5 weeks during exposure to electromagnetic fields. The animals were exposed for 5 weeks to a GSM relay antenna (900 MHz, E eff = 25 V/m) for 24 h per day. The control groups were maintained under the same environmental conditions but protected from the antenna generating the waves.

### 2.2 Beam balance test

The balance beam test was used to assess electromagnetic waves induced sensorimotor deficit. Young rats were tested on a beam (90 cm long by 1.5 cm wide). The balance beam was elevated 80 cm from the floor. Rats were placed in the middle of the beam. The maximal time of each trial is 60 s (sec). Rats underwent a training session during which they had to stay in balance on the beam at three times in each day. The rats were trained at Day-1 (baseline) before exposure and after five weeks of exposure at five consecutive days (from Day+1 to Day +5).

### 2.3 Object recognition test (ORT)

Object recognition task evaluates the rat׳s ability to recognize a novel object in the environment. The test was done directly after 5 weeks of exposure. The ORT is now among the most commonly used behavioral tests for rats. The test was done in spaced box of respective (80–80–40 cm) in which the rats were presented with two similar objects (duplo toys) during the first session, and then one of the two objects is replaced by a new object during a second session. The amount of time taken to explore the new object provides an index of recognition memory. The task procedure consists of three phases: habituation, familiarization, and test phase. In the habituation phase, each animal is allowed freely exploring the open-field area in the absence of objects for 5 min (min). The animal is then removed from the area and placed in its holding cage for 5 min. During the familiarization phase, a single animal is placed in the open-field arena containing two identical sample objects (*A* + *A*), for 5 min. To prevent coercion to explore the objects, rats are released against the center of the opposite wall with its back to the objects. The experimental context is not drastically different during the familiarization and the test phase. After a retention interval (5 min), during the test phase, the animal is returned to the open-field arena with two objects, one is identical to the sample and the other is novel (*A* + *B*). Attention should be paid to the object odors. Thus, the objects should be carefully cleaned before being used for another animal.

### 2.4 Morris water maze test

The behavioral training and testing was conducted in a water maze. The MWM test was conducted in a circular pool 183 cm in diameter and 38 cm tall with a white-painted inner surface. The pool was filled to a depth of 25 cm with water that was maintained at 25 °C. Milk was added to make water opaque. The maze was located in a room containing several visual extra-maze cues for spatial training. A hidden platform (15 cm white circle) was submerged 1 cm below the water surface and placed in the center of one of the quadrant. Each rat was given three trials per day for 7 days to find the hidden platform (training trial). A trial was initiated by randomly placing the rat in one of the four quadrants facing the pool wall; all three quadrants were used once every day for each rat. Each day, the location of the platform was changed into the following quadrant. For each trial, the rat was allowed to swim for a maximum of 60 s to find the platform and upon fail to find the platform the rat was guided to the platform and left on it for 20 s. Escape latency per group was defined as the average time of three quadrant tests on training days. This experiment was done after one week of removing the rats from exposure. Since the feces defecated during the last trial may serve as intra-maze cues, any feces were removed from maze before starting the next trial. After training, all rats transported to their home cage.

### 2.5 Statistical analysis

The values presented in this article are mean ± SEM. ORT was analyzed by analysis of variance (ANOVA) a two-way analysis of variance (ANOVA) followed by the post hoc Bonferroni test for multiple comparisons (MWM). The significant level was a *P*-value less than 0.05.

